# Monocular Initialization for Real-Time Feature-Based SLAM in Dynamic Environments with Multiple Frames

**DOI:** 10.3390/s25082404

**Published:** 2025-04-10

**Authors:** Hexuan Dou, Bo Liu, Yinghao Jia, Changhong Wang

**Affiliations:** Space Control and Inertial Technology Research Center, School of Astronautics, Harbin Institute of Technology, Harbin 150001, China

**Keywords:** visual SLAM, localization, mapping, computer vision

## Abstract

Two-view epipolar initialization for feature-based monocular SLAM with the RANSAC approach is challenging in dynamic environments. This paper presents a universal and practical method for improving the automatic estimation of initial poses and landmarks across multiple frames in real time. Image features corresponding to the same spatial points are matched and tracked across consecutive frames, and those that belong to stationary points are identified using ST-RANSAC, an algorithm designed to detect inliers based on both spatial and temporal consistency. Two-view epipolar computations are then performed in parallel among frames and corresponding features to select the most reliable initialization. The proposed method is integrated with ORB-SLAM3 and evaluated on dynamic datasets for comparative analysis with the baseline. The experimental results demonstrate that the proposed method improves the accuracy of initial pose estimations with the construction of static landmarks while significantly reducing feature extraction scale and computational cost.

## 1. Introduction

Simultaneous localization and mapping (SLAM) is a technique that concurrently estimates poses and generates a map through sensory perception, playing a crucial role in the guidance, navigation, and control (GNC) of autonomous robots operating in unknown environments. Visual-based SLAM has been extensively studied and applied due to the lightweight and cost-effective nature of cameras. The initialization of system states in visual SLAM, involving an initial guess of poses and landmarks, can be achieved through integration with other sensors (IMU, LiDAR, etc.), auxiliary cameras (depth, stereo, etc.), or preset landmarks. Meanwhile, for monocular feature-based visual SLAM without prior information, the initialization is typically performed using structure from motion (SfM), which requires sufficient and correct image feature matching between frames and appropriate camera displacement.

To address measurement errors, mismatching, and features on moving points, modern SLAM systems generally extract excess features for robust initialization. Inlier features consistent with an anticipated motion model are identified, often using statistical methods such as Random Sample Consensus (RANSAC), for multiple-view structure computation. However, in some dynamic environments, features from slow-moving objects may be incorrectly treated as static, leading to erroneous state estimation [1].

To ensure robust monocular initialization, some SLAM algorithms significantly increase feature extraction during bootstrapping to enhance the quantity of stationary features, albeit at the cost of additional computational overheads. Other methods focus on achieving consistent solutions across multiple frames using computationally intensive approaches, which often involve conditional assumptions [2] and require substantial processing time.

In this work, a universal monocular initialization method for dynamic scenes is proposed, which automatically identifies stationary feature pairs and estimates initial pose and landmarks across several frames with a manageable computational burden. The main contributions are as follows:

- The RANSAC algorithm is extended to the discrete time domain, enabling the identification of inlier features with both spatial and temporal consistency. The features corresponding to stationary points are then distinguished from moving ones across consecutive image frames.
- A pipeline is developed to perform automatic initialization using aforementioned features and frames. Two-view epipolar computations between frame pairs are performed in parallel, seeking the most credible estimation of initial poses and map points.
- The proposed initialization method is implemented and integrated into the open-source ORB-SLAM3 framework. Experimental evaluation on dynamic datasets demonstrate that the proposed method achieve superior accuracy in initial pose estimation with map points constructed from static objects while reducing computational time due to the need for fewer extracted features.

This paper is structured as follows. Related works on the initialization of feature-based visual SLAM are briefly surveyed in Section 2. Reviewing conventional two-view estimation, epipolar consistency across multiple frames is discussed, referring to the Spatio-Temporal RANSAC algorithm in Section 3. In Section 4, a pipeline is detailed to estimate initial poses and construct the stationary environment in dynamic scenes with the aforementioned algorithms. To validate the proposed method, dataset experiments are conducted in Section 5, and the experimental results are analyzed in Section 6. The contribution of this paper is concluded in Section 7, with a discussion of future work.

## 2. Related Works

While some systems employ random initial states, most monocular SLAM algorithms rely on 3D perception derived from 2D image information, primarily through the depth estimation of pixels projected onto images [3,4]. Two-view epipolar-based initialization has been extensively applied in many classical SLAM systems [5]. This approach involves estimating the fundamental or homography matrix from matched image features, decomposing the matrix for relative pose with chirality, and triangulating pixel correspondences for landmarks.

With the assumption that matched features are projected from a static environment, this initialization method is challenging in dynamic scenes, where features on moving objects may lead to inconsistency in the statistical epipolar estimation [6]. One solution is to reduce the impact of dynamic features. Regarded as one of state-of-the-art methods with the best performance in dynamic scenes [7], ORB-SLAM3 [8] greatly expands the feature extraction scale during initialization in the codes, improving the robustness of epipolar estimation with an increased number of stationary features. However, the excess feature extraction introduces a heavy computational burden, potentially compromising real-time performance. Additionally, some dynamic features are still misidentified and mixed with stationary ones after RANSAC, resulting in deviated poses and the creation of non-static landmarks.

Another solution is to distinguish and eliminate features on dynamic objects. Semantic-based approaches have gained traction with advancements in data-driven image segmentation. For instance, DynaSLAM [9] provide an influential framework where features on movable objects are double-checked by means of multi-view geometry after CNN-based segmentation, inspiring a number of subsequent studies [10]. However, the instance segmentation efficacy depends on the quality of the pretrained model. Furthermore, achieving real-time performance in semantic segmentation remains a challenge without GPU acceleration [11].

As for geometry-based methods, one of the fundamental theories is multibody epipolar constraint in motion tracking under noise-free conditions. Initialization in actual systems can be deployed using either the iterative computation of multiple hypotheses [12] or prior knowledge of the ego-motion kinematics model [13]. However, the assumption of rigid-body motion for dynamic objects limits their general applicability. Aiming at highly non-grid environments with deformation, the first step of initialization in NR-SLAM [14] assumes that most image innovation comes from camera motion rather than from changing backgrounds, and the estimation is further refined with deformable bundle adjustments in the next step.

Additionally, for most SLAM systems, the primary goal during initialization is motion awareness and exclusion rather than explicit motion tracking, suggesting that these methods could benefit from further optimization for efficiency. For instance, under the assumption of motion consistency around local patches, the dynamic area can be identified and discarded via neighbor-based RANSAC [15] during initialization. The stationary image regions for initialization can also be determined with high confidence in the probabilistic field on Sim(3) manifold [16] via random sampling.

## 3. Stationary Feature Sorting with Spatio-Temporal Consensus

### 3.1. Two-View Epipolar Constraint in Dynamic Environment

**Figure 1:**
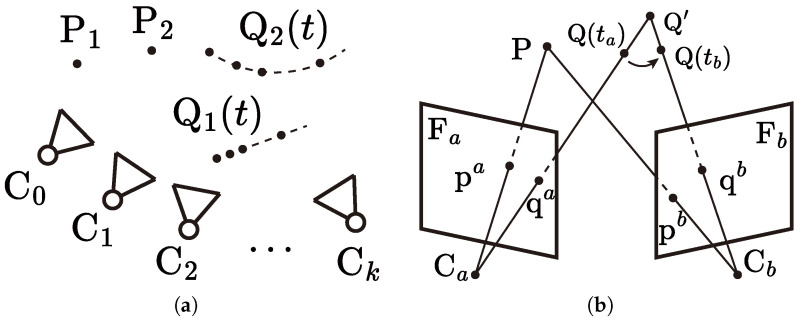
Visual SLAM in dynamic environments. (**a**) Spatial points are observed by consecutive camera centers, where the positions of stationary points $\left\{P_{i}\right\}$ remain fixed, while those of moving points $\left\{Q_{i}\right\}$ vary over time. (**b**) Misidentification of moving points between two views can lead to incorrect epipolar estimation, potentially resulting in ill-initialized states.

As illustrated in Figure 1a, stationary spatial points $P=\{P_{i}\}$ and moving spatial points $Q=\{Q_{i}\}$, with world coordinates $\left\{P_{i}\right\}$ and $\left\{Q_{i}\left(t\right)\right\}$, are observed by a moving camera with camera centers $C=\left\{C\left(t_{0}\right),C\left(t_{1}\right),\ldots ,C\left(t_{k}\right),\ldots \right\}:=\left\{C_{k}\right\}$ at discrete time instances. The corresponding camera poses are represented by the extrinsics $\left\{C_{k}\right\}$. The observed points are projected on the image frame $F_{k}$ of camera center $C_{k}$, where they are represented as image features $P_{k}=\left\{p_{i}^{k}\right\}$ and $Q_{k}=\left\{q_{i}^{k}\right\}$ with homogeneous coordinates $\left\{p_{i}^{k}\right\}$ and $\left\{q_{j}^{k}\right\}$, respectively.

To estimate the initial states of the SLAM system, a RANSAC-based scheme can be applied between camera centers $C_{a}$ and $C_{b}$ using matched features $X_{a}=\left\{x_{i}^{a}\right\}=P_{a}\cup Q_{a}$ and $X_{b}=\left\{x_{i}^{b}\right\}=P_{b}\cup Q_{b}$, seeking epipolar consistency in accordance with either the fundamental matrix $F_{ba}$, satisfying

(1) $$x_{i}^{b⊺}F_{ba}x_{i}^{a}=0,$$

or the homography matrix $H_{ba}$ [17], satisfying

(2) $$\;x_{i}^{b}=H_{ba}x_{i}^{a}.$$

The matrix with the maximum number of consistent matchings and minimal error is estimated using the RANSAC algorithm. The corresponding features in $\left\{x_{i}^{a}\right\}$ and $\left\{x_{i}^{b}\right\}$ satisfying the epipolar constraint are regarded as inliers, thus are considered *spatially consistent* between camera centers $C_{a}$ and $C_{b}$. From this, the relative camera extrinsics $C_{ba}=\left[R_{ba}|t_{ba}\right]$ of $C_{b}$ with regard to $C_{a}$ are computed as the initial pose, and inlier features are triangulated to form initial landmarks.

The feature pairs corresponding to moving points Q, along with mismatched pairs, are expected to be recognized as outliers during RANSAC due to the violation of the epipolar constraint. However, when the displacement between $\left\{Q_{i}\left(t_{a}\right)\right\}$ and $\left\{Q_{i}\left(t_{b}\right)\right\}$ is subtle (e.g., due to a similar motion direction, moderate movement, or non-rigid motion), the corresponding features $Q_{a}$ and $Q_{b}$ may be misidentified as spatially consistent, as illustrated in Figure 1b.

An increase in such misidentified features can lead to significant deviation during the estimation of $F_{ba}$ or $H_{ba}$, resulting in ill-initialized states with drifted pose and some non-stationary landmarks. Therefore, the accuracy of initialization can be improved by eliminating non-stationary features for epipolar matrix estimation.

### 3.2. Temporal Consistency Across Multiple Views

To better distinguish stationary features from moving ones, the epipolar constraint can be checked between multiple pairs of consecutive frames $F=\{F_{k}\}$.

According to the epipolar constraint of Equations (1) and (2), given $F_{ba}$ or $H_{ba}$, spatial consistency can be evaluated with reference to the symmetric transfer error [18], denoted as

(3) $$S\left(x_{i}^{a},x_{i}^{b}\right)=\left\{\begin{array}{c} \;\max(d{\left(x_{i}^{a},x_{i}^{b⊺}F_{ba}\right)}^{2},d{\left(x_{i}^{b},F_{ba}x_{i}^{a}\right)}^{2}),\mathrm{if}\;\mathrm{fundamental}\\ \max(∥x_{i}^{b}-H_{ba}x_{i}^{a}∥,∥x_{i}^{a}-H_{ba}^{-1}x_{i}^{b}∥),\mathrm{if}\;\mathrm{homography} \end{array}\right.$$

where d(x,l) is the distance from a point to a line.

Considering all frame pairs $\left\{\left(F_{a},F_{b}\right)|\forall F_{a},F_{b}\in F,a\neq b\right\}$ within F, the features that are spatially consistent across each pair are referred to as *temporally consistent*, which is represented as

(4) $$S\left(x_{i}^{a},x_{i}^{b}\right)=0,\;\forall F_{a},F_{b}\in F.$$

All features corresponding to stationary spatial point P are expected to be temporally consistent, and they are represented as

(5) $$S\left(p_{i}^{a},p_{i}^{b}\right)<\epsilon ,\;\forall F_{a},F_{b}\in F,\;\forall P_{i}\in P,$$

where ϵ is a threshold considering the measurement error.

For features corresponding to moving spatial points Q, there may be pairs that are misidentified as spatially consistent via RANSAC within the threshold, as discussed in Section 3.1. However, except for possible temporarily fixed points on moving objects, nearly all non-stationary points inherently exhibit spatial inconsistency between certain frame pairs, which is represented as

(6) $$\forall^{\infty }Q_{i}\in Q,\;\exists F_{a},F_{b}\in F,\;s.t.\;S\left(q_{i}^{a},q_{i}^{b}\right)>\epsilon .$$

Compared to moving points Q, stationary points P are more likely to be considered temporally consistent via the traversal of the RANSAC-based spatial consistency check. Consequently, rather than being simply spatially consistent in a single two-view RANSAC process, a spatial point has a much higher likelihood of being stationary if it exhibits temporal consistency across multiple frames.

### 3.3. Spatio-Temporal RANSAC Algorithm

To sort stationary features for improved initialization, temporal consistency can be checked across multiple frames. Similarly to the two-view RANSAC algorithm, numerous random sets of matched features could be checked for epipolar consistency across all or randomly selected pairs of frames. The set of epipolar matrices with the maximum consensus of features would yield the best result for initialization. However, if the algorithm fails to find a consistent solution within the current set of frames, a thorough recalculation is required when a new frame arrives, which can be computationally expensive for real-time SLAM systems.

In this paper, an iterative method is presented to check and record the spatial consistency of features. The temporal consistency of a point is evaluated using the spatial consistency ratio of corresponding features, which is continuously updated as new frames are processed. This method is referred to as Spatio-Temporal RANSAC (ST-RANSAC), and it is detailed in Algorithm 1.
**Algorithm 1** Spatio-Temporal RANSAC at $t_{N}$    **Input:** *M Matched features $\left\{x_{i}^{j}\right\}$ on N+1 frames $\left\{F_{j}\right\}$, with previous counts of being inliers $\left\{m_{i}\right\}$ and outliers $\left\{n_{i}\right\}$*    **Output:** *Stationary features $P_{N}$ on frame $F_{N}$*1:**for** each j∈[0,N−1] **do**2:   Estimate ${\hat{F}}_{jN}$ or ${\hat{H}}_{jN}$ with $\left\{x_{i}^{j}\right\}$, $\left\{x_{i}^{N}\right\}$ by RANSAC3:   **for** each i∈[1,M] **do**4:      Calculate $e=S(x_{i}^{j},x_{i}^{N})$ with ${\hat{F}}_{jN}$ or ${\hat{H}}_{jN}$5:      **if** e<ϵ **then**6:        $m_{i}\leftarrow m_{i}+1$;7:      **else**8:        $n_{i}\leftarrow n_{i}+1$;9:      **end if**10:    **end for**11:**end for**12:**for** each i∈[1,M] **do**13:   Calculate $r_{i}=m_{i}/\left(m_{i}+n_{i}\right)$14:   **if** $r_{i}>r_{\mathrm{th}}$ **then**15:     $P_{N}\leftarrow P_{N}\cup \left\{p_{i}^{N}\right\}$16:   **end if**17:**end for**18:**return** $P_{N}$

Considering the incoming frame $F_{N}$ at $t_{N}$, a spatial consistency check of matched features $X_{N}$ is performed *N* times between $F_{N}$ and all preceding frames $\left\{F_{0},F_{1},\ldots ,F_{N-1}\right\}$ using RANSAC-estimated ${\hat{F}}_{jN}$ or ${\hat{H}}_{jN}$, as detailed in Algorithm 2, for each pair. The counts of inliers and outliers for each feature $x_{i}$ are accumulated as $m_{i}$ and $n_{i}$, respectively, which are inherited from the previous iteration at $t_{N-1}$. Consequently, the ratio $r_{i}=m_{i}/\left(m_{i}+n_{i}\right)$ represents the temporal consistency of feature $x_{i}$ across the frame pairs.

If the ratio $r_{i}$ exceeds a threshold $r_{\mathrm{th}}$, feature $x_{i}$ is considered temporally consistent and thus stationary up to $t_{N}$. Once more than $s_{\mathrm{th}}$ stationary points are recognized at $t_{N}$ by frame $F_{N}$, initialization can be conducted using corresponding features. If the number of stationary points is insufficient, the algorithm will proceed to the next iteration with the incoming frame $F_{N+1}$ at $t_{N+1}$.
**Algorithm 2** Epipolar estimation between $F_{j}$ and $F_{N}$ at $t_{N}$ within *K* iterations    **Input:** *M Matched pairs $D_{Nj}$ between features $\left\{x_{i}^{N}\right\}$ on frame $\left\{F_{N}\right\}$ and $\left\{x_{i}^{j}\right\}$ on frame $\left\{F_{j}\right\}$*    **Output:** *Initial guess of fundamental matrix ${\hat{F}}_{jN}$ or homography matrix ${\hat{H}}_{jN}$*1:**for** each k∈[1,K] **do**2:   Randomly sample 8 pairs of matched feature between $\left\{x_{i}^{N}\right\}$ and $\left\{x_{i}^{j}\right\}$ into set $D_{k}$3:   (1) Estimate ${\hat{F}}_{k}$ with $D_{k}$ using 8-Point algorithm4:   **for** each l∈[1,M−8] **do**5:     Compute fundamental error $e_{l}$ of pair $\left(x_{l}^{N},x_{l}^{j}\right)\in D_{Nj}-D_{k}$ using ${\hat{F}}_{k}$6:     **if** $e_{l}<\epsilon_{\mathrm{th}}$ **then**7:        Accumulate $\epsilon_{\mathrm{th}}-e_{l}$ to score $f_{k}$8:     **end if**9:   **end for**10:   **if** $f_{k}>f_{\max}$ **then**11:     $f_{\max}=f_{k}$, ${\hat{F}}_{\max}={\hat{F}}_{k}$12:   **end if**13:   (2) Estimate ${\hat{H}}_{k}$ with $D_{k}$ using DLT algorithm14:   **for** each l∈[1,M−8] **do**15:     Compute homography error of pair $\left(x_{l}^{N},x_{l}^{j}\right)\in D_{Nj}-D_{k}$ using ${\hat{H}}_{k}$16:     **if** $e_{l}<\epsilon_{\mathrm{th}}$ **then**17:        Accumulate $\epsilon_{\mathrm{th}}-e_{l}$ to score $h_{k}$18:     **end if**19:   **end for**20:   **if** $h_{k}>h_{\max}$ **then**21:     $h_{\max}=h_{k}$, ${\hat{H}}_{\max}={\hat{H}}_{k}$22:   **end if**23:**end for**24:**if** $f_{\max}>h_{\max}$25:   **then return** ${\hat{F}}_{jN}={\hat{F}}_{\max}$26:**else**27:   **return** ${\hat{H}}_{jN}={\hat{H}}_{\max}$28:**end if**

Compared to conventional two-frame-based initialization, the proposed algorithm leverages historical information from all previous frames through the accumulated values of $\left\{m_{i}\right\}$ and $\left\{n_{i}\right\}$. Regarding algorithmic efficiency, the spatial consistency checks between each frame pair (from Line 1 to Line 11 in Algorithm 1) are entirely independent, making it possible to conserve processing time via multithreading or parallel computing. Similarly in Algorithm 2, the estimation of ${\hat{F}}_{k}$ (Line 3 to 12) and ${\hat{H}}_{k}$ (Line 13 to 22) is performed in parallel due to their independence.

## 4. SLAM Initialization with Multiple Frames

With temporally consistent features, two-view or multi-view structure computation can be performed across historical frames. This paper introduces an initialization pipeline that seeks the most credible two-view reconstruction.

### 4.1. Feature Matching Across Multiple Frames

**Figure 2:**
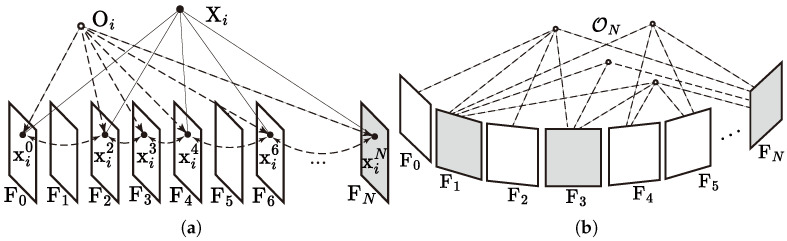
Feature management across multiple frames via object points. (**a**) Matched features corresponding to spatial point $X_{i}$ are linked to object point $O_{i}$, enabling efficient retrieval and association across frames. (**b**) Covisibility between frames is evaluated using object points $O_{N}$ observed on $F_{N}$. Frames sharing the highest number of common observations are selected as candidates for structure computation.

The aforementioned sorting algorithm relies on features being properly matched across multiple frames. However, most extraction algorithms tend to be semi-random during corner detection [19], making it difficult to ensure that features corresponding to the same spatial point appear consistently in successive frames. To effectively look up previous matching, corresponding features across frames can be linked using a data structure referred to as object point, as depicted in Figure 2a.

Similarly to the map points in many SLAM systems, the object point’s structure can be regarded as a candidate landmark with undetermined coordinates. It allows for the efficient association and retrieval of features through their corresponding frames and vise versa. For an incoming frame at $t_{N}$, the feature matching across frames, along with object point maintenance, is detailed in Algorithm 3.
**Algorithm 3** Feature matching across frames at $t_{N}$    **Input:** *M features $X_{N}=\{x_{i}^{N}\}$ on frame $F_{N}$*, with object points O within *N* historical frames $\left\{F_{j}\right\}$    **Output:** *Object points O within N+1 frames*1:**for** each j∈[0,N−1] in reversed order **do**2:   **for** each i∈[1,M] **do**3:     **if** $x_{i}^{N}$ has been matched **then**4:        **continue**5:     **else**6:        Try to find a match for $x_{i}^{N}$ with $x_{*}^{j}$ in $X_{j}$7:        **if** $x_{i}^{N}$ cannot find a match in $X_{j}$ **then**8:          **continue**9:        **else**10:          **if** $x_{*}^{j}$ had been matched and related to $O_{*}$ **then**11:             Relate $x_{i}^{N}$ to $O_{*}$12:          **else**13:             Create $O_{*}$ and relate $x_{i}^{N}$, $x_{*}^{j}$ to $O_{*}$14:             $O\leftarrow O\cup \{O_{*}\}$15:          **end if**16:        **end if**17:     **end if**18:   **end for**19:**end for**20:**return** O

This algorithm ensures that matched features are properly associated via the introduction of object points while avoiding circular matching across frames. As a result, the feature pairs required by Line 2 in Algorithm 1 can be effectively retrieved by means of the management of the object point database O. Additionally, the consistency evaluation of features is transferred to object points for convenience. Concerning the possible computational cost of feature matching across excessive frames, the size of historical frames $\left\{F_{j}\right\}$ can be restricted using a sliding window.

### 4.2. Structure Computing in Parallel

With sufficient stationary points observed at $t_{N}$, two-view structuring can be attempted between $F_{N}$ and multiple preceding frames, aiming for the best initialization with the maximum number of initial landmarks. Frame candidates are determined based on covisibility relationships, as illustrated in Figure 2b. For each stationary object point in $F_{N}$, the preceding frames with the corresponding feature are counted. The frames observing the most stationary object points in common with $F_{N}$ are selected as candidates, with their number adjustable based on the multithreading capability of the processor.

Rather than relying on previously estimated ${\hat{F}}_{iN}$ or ${\hat{H}}_{iN}$ during ST-RANSAC, structure computing for each frame candidate $F_{i}$ is performed using a re-estimated epipolar matrix based solely on sorted stationary object points. Poses and landmarks are then computed for each frame candidate, and the set with the maximum number of triangulated landmarks is selected as the most credible initialization. Similarly to ST-RANSAC, the epipolar re-estimation and structure computation for each candidate frame are completely independent and can be parallelized to enhance computational efficiency.

Compared to conventional methods that search for the best candidate relative to a fixed frame, this pipeline ensures that both frames in the optimal pair are chosen from all historical images. Furthermore, since epipolar estimation using exclusively stationary features is expected to yield greater precision, it becomes feasible to avoid excess feature extraction during initialization, significantly mitigating the computational burden. Consequently, the proposed method has the potential to enhance initialization not only in accuracy but also in efficiency by conserving the scale of feature extraction.

### 4.3. Implementation and Comparison with ORB-SLAM3

**Figure 3:**
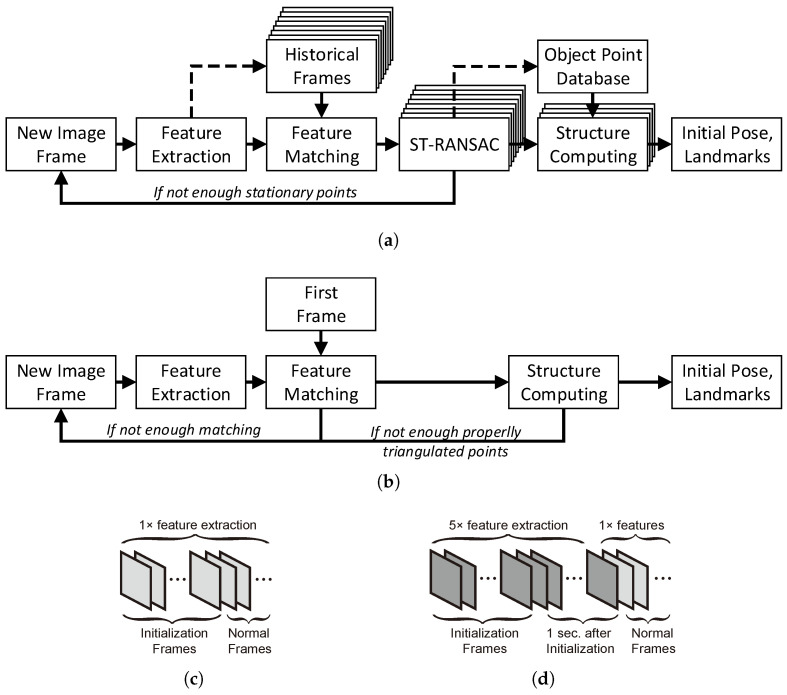
The monocular initialization procedure of (**a**) the proposed method and (**b**) ORB-SLAM3, with the indication of extracted features per frame of (**c**) the proposed method and (**d**) ORB-SLAM3.

The entire initialization pipeline is depicted in Figure 3a and implemented within ORB-SLAM3 [8], a state-of-the-art feature-based SLAM system. After features are extracted from an image, the frame is matched with historical frames to update the object point database. ST-RANSAC is then executed concurrently across multiple threads to distinguish stationary object points in the current frame. Once frame candidates with the highest covisibility are identified, structure computations are performed in parallel. The result with the highest number of triangulated landmarks is accepted as the SLAM system’s initialization, with the current frame referred to as the construction frame and the other as the initial frame.

For comparison, the original monocular initialization procedure in ORB-SLAM3 is shown in Figure 3b. Unlike conventional two-frame initialization, the proposed method fully utilizes historical frame information and conducts multiple structure computations to realize the optimal initial states. The efficiency of both ST-RANSAC and structure computation is ensured through multithreading.

In the original ORB-SLAM2 [20] and DynaSLAM codes, the number of features extracted per uninitialized frame is set to twice that of normal frames, yet initialization failures persist in dynamic scenes. ORB-SLAM3 adopts the same initialization pipeline as ORB-SLAM2 but increases this factor to 5 for robustness in codes, as depicted in Figure 3d. Moreover, this approach extracts additional features in frames following initialization (within 1 s), consuming significant computational resources and compromising real-time performance.

In contrast, the proposed method maintains a consistent number of features across all frames, including uninitialized ones, as depicted in Figure 3c. This consistency ensures steady image processing time for all incoming frames, significantly reducing overall computational time. Additionally, the major extra computation introduced by the proposed method occurs during the parallelized ST-RANSAC stage, which consumes far fewer resources than feature extraction and constitutes only a minor portion of the total processing time.

## 5. Experiments

### 5.1. Dataset Experiments

Dataset experiments were conducted to compare the initialization efficacy of the baseline ORB-SLAM3 and the proposed method. The popular semantic-masking method designated for dynamic environments, DynaSLAM, was also compared for reference despite its inefficiency. Selected monocular sequences from the TUM [21] and Bonn [22] datasets, which include dynamic scenes, were used for evaluation.

The experiments were performed on an *Intel Core i7-12700K* (Intel, Santa Clara, CA, USA) desktop computer without GPU acceleration. The base number of extracted features per frame was set to 1000 for all methods according to the resolution. The first 100 images of each sequence were processed by the SLAM systems, attempting to compute initial poses and map points. In the proposed method, frame tracking after initialization follows the same pipeline as ORB-SLAM3 to ensure a fair comparison. Keeping pace with ORB-SLAM3, the multiplier of features during initialization in DynaSLAM was augmented from the original 2 to 5 to prevent failure. For some sequences, several images containing only stationary backgrounds were skipped at the beginning to focus on dynamic scenes.

The accuracy of the estimated keyframe poses immediately following the initialization was assessed. Since scale ambiguity exists, the Sim(3) transformation was applied using the Umeyama algorithm to align the keyframes with ground truth. The translations of the root mean square error of the absolute trajectory (RMS ATE) and relative poses (RMS RPE) over the initialization were calculated to compare the accuracy of initial poses. For each sequence, multiple experiments were conducted with both methods, and the initialization metrics featuring median RMS ATE are listed in Table 1. The peak time during initialization and the mean tracking time for all frames were recorded to evaluate computational efficiency. Additionally, the frame pair of the initial frame and the construction frame, automatically selected for two-view reconstructions during initialization, is reported for reference.

In the proposed method, the error threshold ϵ introduced in Section 3.2 was set based on the $\chi^{2}$ test at 95%. The temporal consistency threshold $r_{\mathrm{th}}$ introduced in Section 3.3 was set to 0.6. The threshold for the number of stationary object point observations $s_{\mathrm{th}}$ introduced in the same section was set to 50.

### 5.2. Practical Experiments

To further evaluate the proposed method, practical experiments were conducted to compare the initialization efficacy in an outdoor environment. An unmanned ground vehicle (UGV) was remote-controlled to pass through the Science Park campus of Harbin Institute of Technology, capturing monocular image sequences containing moving people and vehicles with an integrated Intel D435i camera (Intel, Santa Clara, CA, USA) at 640×480@30fps. The ComNav M100 system offered RTK GNSS ground truth at 10Hz, with three-dimensional positioning precision within 2cm.

In accordance with dataset experiments, the first 100 images in each of the five dynamic scenes were processed on the aforementioned computer using the SLAM systems repeatedly, attempting to estimate initial poses and map points. The base number of extracted features per frame was set to 1500 for all methods, and the features during initialization were doubled in the proposed method to adapt to the outdoor environment. The other hyperparameters were set to the same values mentioned in the dataset experiments. The metrics of experimental results featuring median RMS ATE are listed in Table 2.

## 6. Discussion

### 6.1. Stationariness of Initial Map Points

Pruning dynamic features, the proposed method successfully constructed static parts of the environment into initial map points.

**Figure 4:**
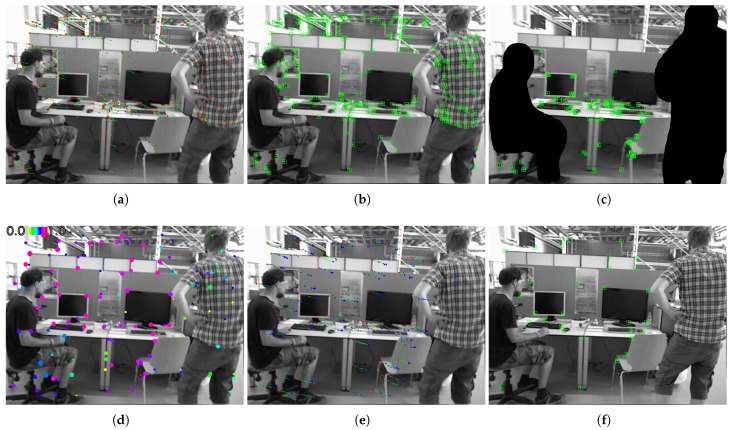
Features and map points during monocular initialization in sampled frames from the sequence freiburg3_walking_xyz. In the original ORB-SLAM3 (extracting 5×1000 features per frame), (**a**) candidate features for initialization and (**b**) initial map points on the 18th construction frame are shown. The initial map points on the 16th construction frame of DynaSLAM (extracting 5×1000 features per frame) are shown in (**c**) for reference. Meanwhile, for the proposed method (extracting 1000 features per frame) of the same frame, (**d**) the spatial consistency ratio is calculated with ST-RANSAC after matching and updating object points, and (**e**) the stationary features are then sorted as candidates for initialization. (**f**) The initial map points on the 22nd construction frame in the proposed method are shown for comparison. Images are adapted from the TUM dataset.

The examples of candidate features for initialization, along with the resulting initial map points, are illustrated in Figure 4, and they are sampled from the widely compared sequence freiburg3_walking_xyz in the TUM dataset.

In ORB-SLAM3, the candidate matches in the 18th construction frame are shown in Figure 4a, which contain a significant number of features on the right, indicating a moving person. Many non-stationary points are subsequently triangulated from these features, resulting in a deviated initial pose and misleading the subsequent localization, as illustrated in Figure 4b.

In the proposed method, all observed object points in the 18th frame are marked in Figure 4d, with the spatial consistency ratio represented in diverse colors and observation counts indicated by different point sizes. The features on the right indicating a moving person exhibit noticeably lower spatial consistency ratios regardless of the observation frequency. The object points sorted with the spatial consistency ratio $r_{\mathrm{th}}$ are shown in Figure 4e as candidate features, where blue points represent the matches in preceding frames. Subsequently, later on in the 25th construction frame of the proposed method, similar stationary features are used to triangulate the initial map points, which are shown in Figure 4f.

The 16th construction frame in DynaSLAM is shown in Figure 4c for reference. Compared with semantic-based masking, the proposed method can achieve comparable mapping efficacy in eliminating dynamic points while tremendously conserving computational resources and processing time.

**Figure 5:**
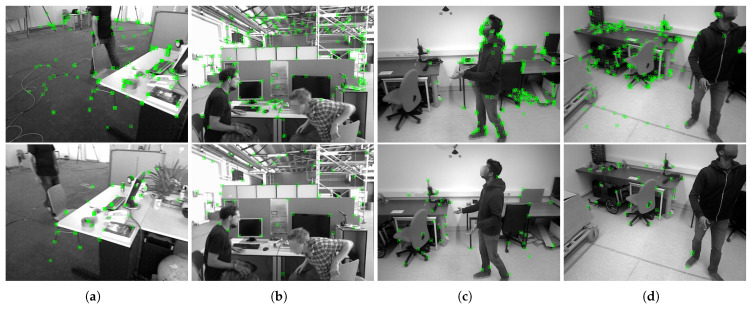

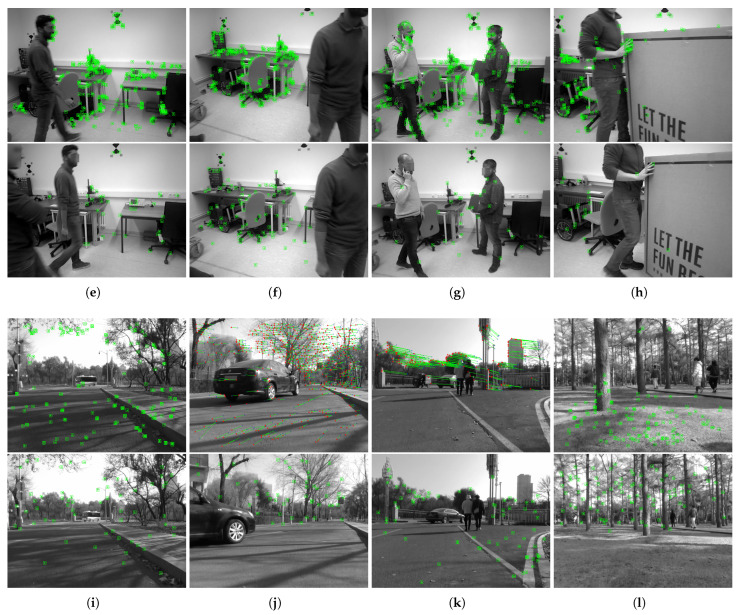
Comparison of initial map points on the construction frames of respective methods in sequence (**a**) Freiburg2_desk_with_person and (**b**) Freiburg3_walking_halfsphere from the TUM dataset; (**c**) Balloon, (**d**) Balloon2, (**e**) Crowd, (**f**) Crowd2, (**g**) Crowd3, and (**h**) Moving_obstructing_box2 from the Bonn dataset; and (**i**) Scene 1, (**j**) Scene 2, (**k**) Scene 3, and (**l**) Scene 4 in practical experiments, with ORB-SLAM3 on the top and the proposed method on the bottom.

Further comparisons of initial map points in other sequences are shown in Figure 5, which are sampled from the respective construction frames. These examples demonstrate that the proposed method effectively separates stationary features from moving ones in dynamic scenes, prompting the construction of map points on fixed objects for better initialization.

### 6.2. Efficacy of Initial Pose Estimation

The proposed method tracks initial poses with better accuracy while conserving processing time by requiring the extraction of fewer features.

The experimental results in Table 1 show that the proposed method consistently [“achieves” or “brings”] lower localization errors compared to the baseline across nearly all sequences, with up to 65.15% less RMS ATE and 67.19% less RMS RPE in sequence freiburg3_walking_xyz. One of the exceptions occurs in the sequence Balloon2, where the RMS ATEs of the two methods are closely comparable with a disparity of 3.17%, while the proposed method still achieves a 29.86% reduction in RMS RPE. The other exception occurs in Scene 4 of the practical experiment, where the baseline method covers a minor part of the trajectory. As for DynaSLAM, the proposed method generally achieves comparable localization accuracy and outperforms it in many sequences.

In additional to accuracy, the proposed method also improves efficiency. The mean tracking time of all 100 frames around the initialization is reduced across almost all sequences. The peak processing time, which occurs at the respective construction frame to perform two-view reconstruction, is generally lowered in most sequences and up to 58.50% in sequence freiburg3_walking_halfsphere.

**Figure 6:**
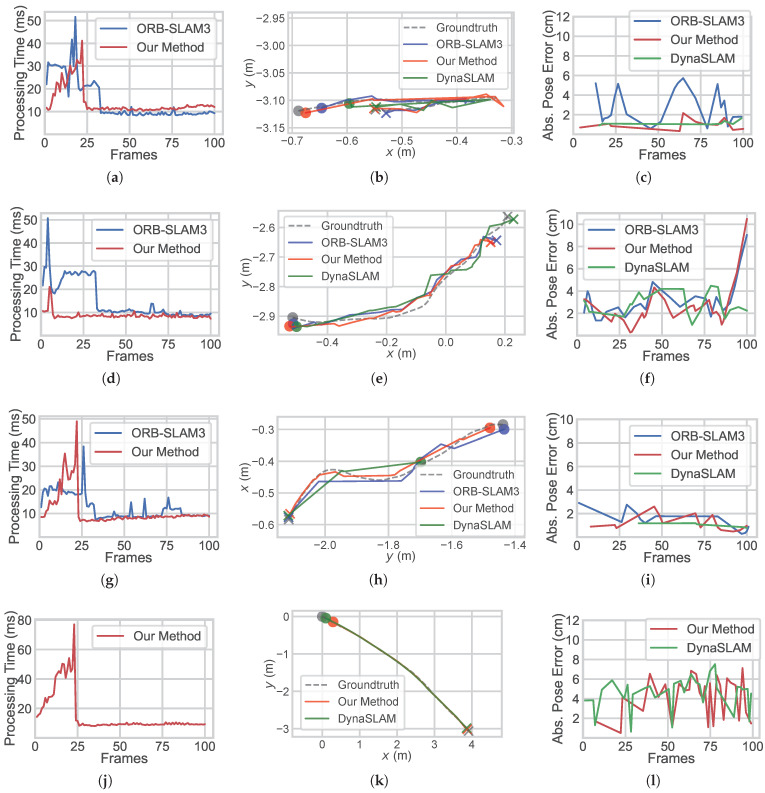

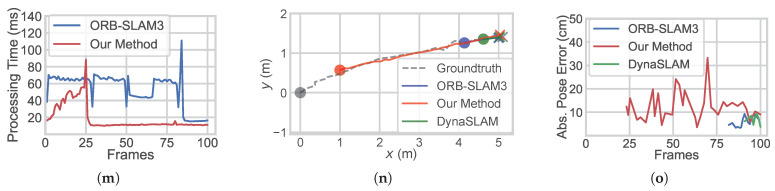
Comparison of metrics in ORB-SLAM3, DynaSLAM, and our method. (**a**) Processing time of frames, (**b**) trajectories, and (**c**) absolute pose error distributions over initialization in the sequence freiburg3_walking_xyz are shown, together with (**d**–**f**) in sequence freiburg3_walking_halfsphere, (**g**–**i**) in sequence moving_obstructing_box, and (**j**–**l**) in Scene 3 and (**m**–**o**) in Scene 4 of the practical experiments.

Plotted by EVO [23], the key frame trajectories of some sequences are shown in Figure 6 as examples, alongside the processing time per frame and absolute pose error (APE) distribution. In the highly misguiding sequence freiburg3_walking_xyz, where initialization is procrastinated in all methods, the proposed method achieves impressive trajectory accuracies with reduced processing time. In the less misguiding sequence freiburg3_walking_halfsphere, where initialization is expeditious in all methods, the proposed method achieves a noticeably reduced processing time, together with the promotion of trajectory accuracy.

In sequence moving_obstructing_box with dynamics that step away from the camera, all methods realized good initialization, implying the limited promotion of the proposed method in such cases.

It is indicated that, with the help of stationary feature sorting, the proposed method realizes more accurate initial poses in dynamic scenes while reducing computational time.

### 6.3. Robustness of Initialization

While requiring the extraction of fewer features, the proposed initialization method is more robust than the others.

After repeated attempts, ORB-SLAM3 or DynaSLAM fails to perform initialization in dataset sequences Crowd, Crowd2, Crowd3, and Person_tracking2. The baseline ORB-SLAM3 is also challenged in Scene 2, Scene 3, and Scene 4 of the outdoor dynamic environment, as shown in Figure 5 and Figure 6. Meanwhile, the proposed method completes initialization in all sequences and scenes within multiple trials. In addition to stationary feature sorting, the robustness of the proposed method is speculated to be ensured by parallel structuring, which automatically compares and selects the frame pair with the most credible matches to perform the initialization.

The success rates of initialization in different datasets and practical experiments are compared in Table 3. The results demonstrate that the proposed method achieves consistently high success rates across a range of scenarios involving various dynamic objects. However, it is noteworthy that rapid ego-motion involving violent rotations remains a significant challenge for the proposed method. It is indicated that increasing the number of features extracted during initialization contributes to the generation of more map points, which in turn enhances the accuracy and robustness of the initialization process. Therefore, increasing the number of extracted features can further improve the performance of the proposed method, albeit at the expense of increased computational overhead.

## 7. Conclusions

This paper introduces a geometry-based method for automatically computing initial poses and landmarks for real-time monocular SLAM in dynamic scenes. With the proposed ST-RANSAC algorithm, stationary features are distinguished from moving ones for more accurate initialization. Through the data structure termed the object point, the parallel processing of epipolar estimation and triangulation is performed to ensure the timeliness of the initialization pipeline. Our experimental results demonstrate that the proposed method surpasses the baseline in both initial pose estimation accuracy and frame processing efficiency.

A key advantage of the proposed method is its ability to maintain steady computational costs for image processing by avoiding the need to extract additional features during initialization. This feature is particularly significant for SLAM algorithm designs in hardware-constrained platforms or specialized chips. While optimized for dynamic environments, this method also shows promise for improving initialization in static scenes. By automatically selecting frame pairs with the highest covisibility, it proves to be potentially advantageous in challenging scenarios, such as sequences with featureless, blurred, or distorted images or low-parallax viewpoints.

Furthermore, during initialization, the identification of temporal consistency for object points could guide feature extraction in subsequent frames, concentrating on stationary areas. This approach might enhance the density and quality of map points constructed on static objects, which is a potential direction for further research.

## Figures and Tables

**Table 1:** Dataset experimental results and comparison.

Dataset	Sequence	Skipped Images	Method	Init. Frame Pair	Peak Init. t. (ms)	Mean Track t. (ms)	RMS ATE (cm)	RMS RPE (cm)
TUM	fr2 desk with person	1060	ORB3 *	25th, 33rd	46.010	14.215	0.452	0.729
			MOD *	7th, 9th	**40.038**	**10.978**	0.397	**0.567**
			Dyna *	1st, 13th	869.127	842.361	**0.341**	0.637
	fr3 walking xyz	-	ORB3	14th, 18th	51.663	14.811	3.265	5.193
			MOD	5th, 22nd	**41.196**	**14.153**	**1.138**	**1.704**
			Dyna	1st, 16th	936.505	866.935	1.150	1.735
	fr3 walking rpy ^†^	-	ORB3	21st, 30th	55.965	**16.152**	1.361	1.414
			MOD	23th, 33th	**50.963**	18.678	**1.307**	**1.253**
			Dyna	21st, 30th	927.457	855.352	1.427	1.713
	fr3 walking halfsphere	-	ORB3	1st, 4th	50.712	15.219	3.650	2.484
			MOD	2nd, 5th	**21.048**	**8.692**	3.228	**1.732**
			Dyna	1st, 4th	869.516	837.786	**2.965**	2.614
Bonn	Balloon	-	ORB3	1st, 7th	43.038	14.449	2.810	7.189
			MOD	16th, 24th	**38.904**	**11.723**	**1.467**	**3.586**
			Dyna	1st, 35th	861.660	843.233	3.990	8.511
	Balloon2	-	ORB3	1st, 9th	39.418	14.179	2.931	12.086
			MOD	4th, 10th	**29.473**	**8.572**	3.027	**8.477**
			Dyna	1st, 32th	881.597	843.639	**2.223**	19.521
	Balloon tracking	280	ORB3	1st, 16th	**40.395**	11.920	21.857	14.458
			MOD	10th, 23rd	51.358	**11.495**	8.396	**11.765**
			Dyna	1st, 26th	864.395	843.188	**6.951**	13.419
	Balloon tracking2	200	ORB3	41st, 45th	**22.181**	**9.023**	4.207	11.710
			MOD	44th, 51st	47.049	14.493	3.289	**11.191**
			Dyna	67th, 70th	870.908	838.281	**2.051**	12.102
	Crowd	230	ORB3	1st, 6th	58.724	17.714	2.752	2.844
			MOD	1st, 16th	**32.468**	**9.618**	**2.314**	**2.407**
			Dyna	Failed	–	–	–	–
	Crowd2 ^†^	-	ORB3	1st, 13th	40.510	12.479	1.110	9.976
			MOD	6th, 10th	**36.449**	**10.346**	**1.011**	**9.373**
			Dyna	Failed	–	–	–	–
	Crowd3	30	ORB3	1st, 4th	48.943	12.375	1.291	3.987
			MOD	4th, 10th	**27.137**	**8.412**	**0.754**	**3.050**
			Dyna	Failed	–	–	–	–
Bonn	Moving nonobstructing box	250	ORB3	1st, 13th	41.738	13.051	2.974	**7.201**
			MOD	8th, 14th	**40.543**	**9.230**	2.682	8.717
			Dyna	1st, 33th	884.943	857.958	**1.712**	17.789
	Moving nonobstructing box2	470	ORB3	1st, 6th	**36.212**	11.934	3.615	5.744
			MOD	23rd, 37th	47.308	**11.450**	**2.717**	**5.523**
			Dyna	1st, 13th	877.393	842.329	2.769	9.729
	Moving obstructing box	300	ORB3	1st, 26th	**38.401**	12.602	1.608	13.985
			MOD	8th, 22th	48.958	**10.982**	1.326	**12.076**
			Dyna	37th, 70th	880.056	841.287	**1.025**	25.772
	Moving obstructing box2	400	ORB3	1st, 3rd	55.763	12.576	4.350	6.197
			MOD	7th, 15th	**39.013**	**12.531**	**2.259**	**4.518**
			Dyna	1st, 5th	869.905	832.819	2.476	9.121
	Person tracking ^‡^	10	ORB3	1st, 10th	**55.084**	13.528	3.422	3.589
			MOD	25th, 37th	55.421	**12.889**	1.727	3.612
			Dyna	1st, 13th	916.646	860.498	0.938	6.470
	Person tracking2	40	ORB3	Failed	–	–	–	–
			MOD	22nd, 24th	47.495	9.875	3.196	2.457
			Dyna	Failed	–	–	–	–

* ORB3: Original ORB-SLAM3 system. MOD: The modified system with the proposed initialization method. Dyna: DynaSLAM system. ^†^ The feature extraction number during initialization is doubled to 2000 in the modified system. ^‡^ Tracking failure occurs after around the 50th frame in DynaSLAM, and thus, the corresponding RMSE is not comparable.

**Table 2:** Practical experimental results and comparison.

Scene	Method	Init. Frame Pair	Peak Init. t. (ms)	Mean Track t. (ms)	RMS ATE (cm)
1	ORB3	1st, 20th	89.969	17.700	4.202
	MOD	4th, 21st	**77.773**	**15.729**	**4.011**
	Dyna	1st, 20th	905.595	859.108	4.026
2	ORB3	Failed	–	–	–
	MOD	12th, 14th	**57.868**	**13.692**	**7.114**
	Dyna	1st, 35th	898.462	861.564	9.001
3	ORB3	Failed	–	–	–
	MOD	8th, 23rd	**77.083**	**15.478**	**4.615**
	Dyna	1st, 7th	909.335	857.531	4.799
4	ORB3	82nd, 84th	111.027	53.391	**6.185**
	MOD	24th, 25th	**88.046**	**18.804**	13.729
	Dyna	91st, 95th	930.409	881.395	7.073
5	ORB3	1st, 26th	74.865	18.443	6.558
	MOD	3rd, 20th	**70.303**	**14.951**	**5.429**
	Dyna	1st, 43rd	894.833	864.254	5.599

**Table 3:** Success rate in experiments.

Scenario	Number of Sequences	Method	Trials	Success Rate	Challenging Sequence
TUM dataset	4	ORB3	20	95%	Walking RPY
		MOD	20	95%	Walking RPY
		Dyna	20	100%	–
Bonn dataset	13	ORB3	65	92.31%	Person Tracking 2
		MOD	65	95.38%	Balloon Tracking 2, Crowd 2
		Dyna	65	69.23%	Crowd 1–3, Person Tracking 2
Practical experiment	5	ORB3	25	60%	Scene 2, 3
		MOD	25	92%	Scene 2
		Dyna	25	88%	Scene 2

## Data Availability

Data are contained within the article or Supplementary Materials.

## Supplementary Materials

The following supporting information can be downloaded at https://www.mdpi.com/article/10.3390/s25082404/s1. Video S1: Short demo.

## Abbreviations

The following abbreviations are used in this manuscript:

APE	Absolute pose error;
ATE	Absolute trajectory error;
DLT	Direct linear transform;
GNC	Guidance, navigation, and control;
GNSS	Global navigation satellite system;
IMU	Inertial measurement unit;
LiDAR	Light detection and ranging;
RANSAC	Random Sample Consensus;
RMS	Root mean square;
RPE	Relative pose error;
RTK	Real-time kinematic positioning;
SLAM	Simultaneous localization and mapping;
ST-RANSAC	Spatio-Temporal Random Sample Consensus;
UGV	Unmanned ground vehicle.
